# Four-way regulation of mosquito yolk protein precursor genes by juvenile hormone-, ecdysone-, nutrient-, and insulin-like peptide signaling pathways

**DOI:** 10.3389/fphys.2014.00103

**Published:** 2014-03-20

**Authors:** Immo A. Hansen, Geoffrey M. Attardo, Stacy D. Rodriguez, Lisa L. Drake

**Affiliations:** ^1^Department of Biology, New Mexico State UniversityLas Cruces, NM, USA; ^2^Institute for Applied Biosciences, New Mexico State UniversityLas Cruces, NM, USA; ^3^Molecular Biology Program, New Mexico State UniversityLas Cruces, NM, USA; ^4^Department of Epidemiology of Microbial Disease, Yale School of Medicine, Yale UniversityNew Haven, CT, USA

**Keywords:** mosquito, vitellogenesis, insulin, juvenile hormone, ecdysone, target of rapamycin, yolk proteins

## Abstract

Anautogenous mosquito females require a meal of vertebrate blood in order to initiate the production of yolk protein precursors by the fat body. Yolk protein precursor gene expression is tightly repressed in a state-of-arrest before blood meal-related signals activate it and expression levels rise rapidly. The best understood example of yolk protein precursor gene regulation is the *vitellogenin-A* gene (*vg*) of the yellow fever mosquito *Aedes aegypti*. *Vg-A* is regulated by (1) juvenile hormone signaling, (2) the ecdysone-signaling cascade, (3) the nutrient sensitive target-of-rapamycin signaling pathway, and (4) the insulin-like peptide (ILP) signaling pathway. A plethora of new studies have refined our understanding of the regulation of yolk protein precursor genes since the last review on this topic in 2005 (Attardo et al., 2005). This review summarizes the role of these four signaling pathways in the regulation of *vg-A* and focuses upon new findings regarding the interplay between them on an organismal level.

## Introduction

### Mosquito species use two divergent reproductive strategies

Mosquito species can be divided into two groups according to their reproductive strategy. Autogenous mosquitoes do not require an initial blood meal and produce their first batch of eggs relying solely on nutrients accumulated during their larval phase. Vitellogenesis, the production of yolk by the fat body for deposition in developing oocytes, begins a few hours after eclosion of autogenous adult females. Subsequent egg batches are dependent upon the energy and nutritional building blocks derived from vertebrate blood.

In contrast, anautogenous mosquitoes require an obligatory blood meal to produce their first batch of eggs. In these species, vitellogenesis is tightly repressed until blood meal-associated signals activate signaling cascades in involved organs and tissues. Most mosquito species fall into one of these two categories. However, there are exceptions to this rule. For example, *Aedes atropalpus* has both autogenous and anautogenous strains. Most of the human disease-transmitting species fall into the anautogenic category. The underlying reason for this is that the necessity for blood by anautogenous mosquitoes drives increased interaction between vector and host making them inherently better disease vectors. In addition, the fact that they feed earlier gives the parasites more time to finish the extrinsic part of their life cycle and become infective within the vector. Autogeny vs. anautogeny is discussed in more detail in Attardo et al. (2005).

### Yolk protein precursor proteins are essential for mosquito reproduction

Insect oocytes are loaded with yolk proteins during the process of vitellogenesis. Yolk proteins provide the essential nutrients required for embryonic development. Mosquitoes produce yolk protein precursors (YPPs) exclusively in the fat body, the insect analog of the vertebrate liver. The YPPs are secreted into the hemolymph and taken up by the developing oocytes via receptor-mediated endocytosis (Sappington et al., 1996). Several yolk protein genes from *Ae. aegypti* have been independently cloned and characterized (Deitsch and Raikhel, 1993; Cho et al., 1999; Sun et al., 2000). The publication of the annotated *Ae. aegypti* genome sequence in 2007 (Nene et al., 2007) facilitated the identification of all potential yolk protein genes *in silico*. A detailed RNA-Seq comparison of genes differentially expressed by the *Ae. aegypti* fat body before and after a blood meal revealed that two genes encoding vitellogenic cathepsin B, three genes encoding vitellogenins (vitellogenin A, B, C), and three genes encoding vitellogenic carboxy-peptidases are up-regulated several hundredfold in the female fat body 24 h after a blood meal (Price et al., 2011). Together with three lesser expressed vitellin membrane proteins, yolk proteins account for more than a third of all messenger RNAs at this time point which represents the pinnacle of vitellogenesis. The abundance of YPP associated gene transcripts reflects the massive scale of protein synthesis the fat body performs during this process.

### Signaling pathways in the fat body

**Figure 1:**
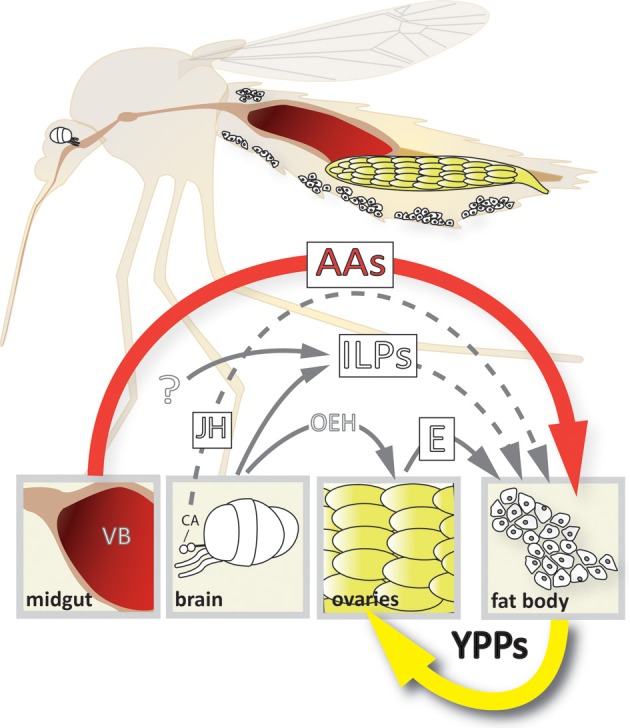
**Model of the 4-way regulation of YPP gene expression in *Ae. aegypti***. The upper part of the scheme depicts a longitudinal section showing the relative position of the different organs involved. Organ and cell size is not up to scale! The lower part shows the different signals involved and their origins. AAs, amino acids; CA, corpora allata; E, ecdysone; ILPs, insulin-like peptides; JH, juvenile hormone; OEH, ovary ecdysteroidogenic hormone; VB, vertebrate blood; YPPs, yolk protein precursors.

Several signaling pathways regulate the transition of the fat body from the previtellogenic state-of-arrest to vitellogenic YPP synthesis. Our understanding of the interplay between these pathways has grown significantly in the last few years (see Figure 1). In this review we summarize the latest findings on each of the following topics: juvenile hormone, ecdysone, nutrient, and insulin-like peptide signaling pathways in mosquitoes, the roles they play in regulating vitellogenic gene expression in fat body trophocytes and the crosstalk that occurs between these signaling pathways on an organismal level.

## Juvenile hormone

### What is it?

Juvenile hormone (JH) is a sesquiterpenoid that regulates insect development, reproduction and other processes in basically all insect species. Different groups of insects use different forms of JH, for instance mosquitoes use JH III (Clements, 1992).

### Where is it made?

JH is synthesized by the corpora allata, a pair of specialized glands that are attached to the brain through the nervus corpora cardiaca and are in close proximity to the aorta.

### How is JH synthesis regulated?

JH levels in the hemolymph and target tissues are thought to be regulated primarily by the rate of synthesis in the corpora allata. JH synthesis is controlled by peptide hormones that reach the glands through the hemolymph or by direct neural connections. Peptide hormones that stimulate JH synthesis are called allatotropins, those that inhibit JH synthesis are termed allatostatins (Gade et al., 1997). Signaling activity of JH is also regulated by proteins that facilitate its transport or breakdown in the hemolymph. Proteins promoting JH hydrolysis include JH esterase and JH epoxide hydrolases (Lassiter et al., 1994, 1995, 1996; Edgar et al., 2000; Bai et al., 2007), while JH-binding proteins facilitate the transport of this lipophilic molecule in the hemolymph (Prestwich et al., 1996). The rate of JH synthesis in newly eclosed female mosquitoes is in close correlation with their nutritional status (Noriega, 2004).

### Mode-of-action

**Figure 2:**
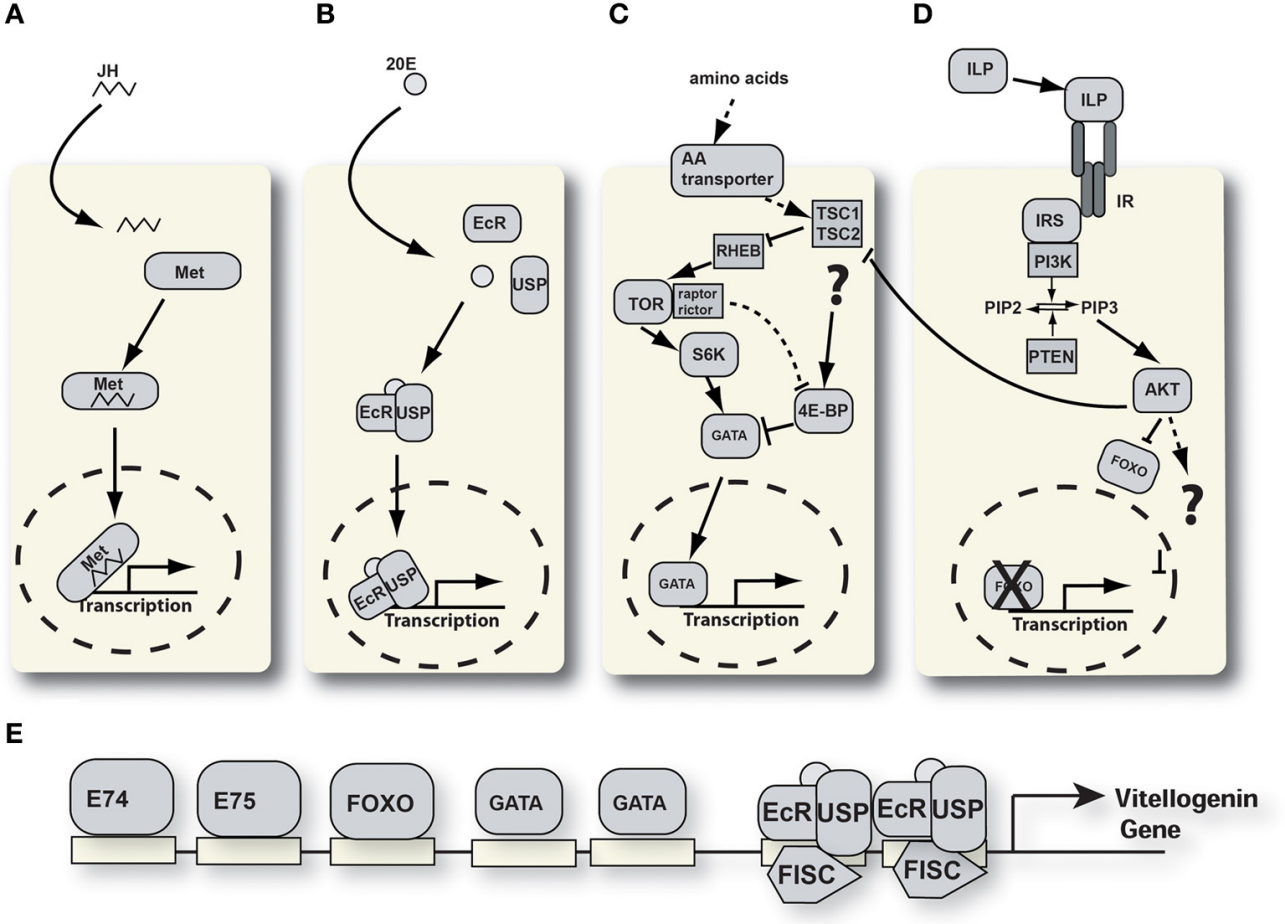
**Signaling pathways involved in YPP regulation**. **(A)** Juvenile hormone signaling pathway. JH, juvenile hormone; Met, methoprene tolerant. **(B)** Ecdysone signaling pathway. 20E, 20 hydroxyecdysone; EcR, ecdysone receptor; USP, ultraspiracle. **(C)** Nutrient signaling pathway. 4E-BP, 4E-binding protein; TSC, tuberous sclerosis complex; RHEB, RAS homologue enriched in brain; S6K, S6 kinase; TOR, target of rapamycin. **(D)** Insulin-like peptide signaling pathway. AKT, protein kinase B; FOXO, forkhead box protein O; ILP, insulin-like peptide; IRS, insulin receptor substrate; PI3K, phosphati dylinositide 3-kinase; PIP2, phosphatidylinositol 4,5-bisphosphate; PIP3, phosphatidylinositol (3,4,5)-triphosphate; PTEN, phosphatidylinositol-3,4,5-trisphosphate 3-phosphatase. **(E)** Schematic of the vg-A promoter with transcription factor binding sites.

See Figure 2A—For several decades, efforts to identify a JH-receptor were unsuccessful. However, in recent years the understanding of JH's mode-of-action has grown significantly (Riddiford, 2012; Jindra et al., 2013). While there are still many open questions, there is growing evidence that the Methoprene-tolerant (Met) protein (and in *Drosophila* also its paralog germ-cell-expressed) acts as intracellular JH-receptor. In complex with other proteins these receptors form active complexes that bind JH-response elements in the DNA and regulate gene transcription (Li et al., 2011; Zou et al., 2013).

### How does JH regulate mosquito YPP expression?

As its name suggests, one of the functions of JH during early postembryonic development is to prevent insect larvae from entering metamorphosis during the earlier larval molts. Another important role of JH is the regulation of insect reproduction. JH plays a central role in the regulation of *YPP* genes in mosquito females (Zou et al., 2013). After eclosion, females of anautogenous mosquito species enter a 3 day maturation period during which they do not blood-feed. Once this period is complete, the now “competent” females enter a state-of-arrest during which vitellogenic activity by the fat body is halted. The state-of-arrest is only broken when a blood meal is taken. Autogenous mosquitoes express *YPP* genes in the fat body earlier and without a blood meal stimulus; for example the vitellogenin-1 gene of *Culex tarsalis* is strongly expressed 24 h after eclosion (Provost-Javier et al., 2010). The ability or inability of an individual anautogenous mosquito to overcome the state-of-arrest and activate YPP gene expression is pre-established by its individual JH exposure history which in turn is correlated with the level of nutrients acquired during the larval phase (Noriega, 2004). Groups of *Ae. aegypti* larvae that are raised under severe nutritional restriction and overcrowded conditions give rise to significantly smaller adults compared to well-nourished under-crowded groups. Such small-sized *Ae. aegypti* females require more than one blood meal to complete vitellogenesis and develop a first batch of eggs (Shiao et al., 2008). This phenotype is due to the altered endocrinology of small mosquitoes; specifically these mosquitoes have lower JH levels during the 3 day maturation period which results in the delayed onset of vitellogenesis (Noriega, 2004). Treatment of small mosquitoes with JH immediately after eclosion results in recovery of the nutrient-signaling machinery (see Nutrient signaling) in the fat body, timely vitellogenesis and the successful development of a batch of eggs after only one blood meal.

Therefore, the role that JH plays in the regulation of YPP expression in the fat body is most likely a preparatory one. Managing JH levels is critical for mosquitoes to regulate and coordinate their nutrient reserves with their reproductive cycle. This hormonally mediated balance avoids nutrient shortages and optimizes egg production.

It must be emphasized that the effects of JH on vitellogenesis and egg development are in no way restricted to the mosquito fat body. For example, a study by Clifton and Noriega showed that JH levels determine the fate of individual ovarian follicles in *Ae. aegypti* thereby determining the final egg numbers that are produced in a given gonotropic cycle (Clifton and Noriega, 2012).

## Ecdysone

### What is it?

Ecdysteroids are insect steroid hormones which, like JH, are key regulators of molting as well as reproduction in all holometabolous insects. Mosquitoes use the ecdysteroid ecdysone which is thought to be converted to its more bioactive form, 20-hydroxyecdysone (20E), in the fat body and other peripheral tissues, as described in *Drosophila* (Raikhel et al., 2002; Gilbert, 2004; Rewitz et al., 2006).

### Where is ecdysone made?

During larval development ecdysone is synthesized by the prothoracic glands. However, these glands degenerate and disappear rapidly after adult emergence (Clements, 1992). In adult female mosquitoes ecdysone is produced by the epithelial cells of the ovarian follicle in response to blood meal-derived signals (Hagedorn, 1982, 1989; Raikhel et al., 1999).

### How is ecdysone synthesis regulated?

During larval development a peptide hormone, prothoracicotropic hormone (PTTH), is released from the brain and stimulates ecdysone synthesis in the prothoracic gland by activating the RAS/ERK pathway (Gilbert et al., 2002; Rewitz et al., 2009). In adult females a different peptide hormone, the ovary ecdysteroidogenic hormone (OEH), substitutes the role of PTTH. OEH is produced in neurosecretory brain cells and stored in the corpora cardiaca (Brown and Cao, 2001). Upon blood feeding, stretch receptors within the midgut wall trigger a signal to the brain and OEH is released into the hemolymph of the mosquito (Hagedorn et al., 1975). OEH stimulates the follicle cells of the ovaries to synthesize and release ecdysone (Dhara et al., 2013). Four hours post-blood meal (PBM) ecdysone hemolymph titers are slightly elevated. Levels continue to rise reaching a peak at 18–24 h PBM and decline afterwards (Hagedorn et al., 1975). OEH is a prominent regulator of ecdysone synthesis, but is by no means the only factor involved. Insulin-like peptide signaling also plays a role (see below).

### Mode-of-action

See Figure 2B, 20E activates transcription of target genes by binding to a cytoplasmic receptor. The ecdysone receptor is a heterodimeric complex consisting of two proteins, the ecdysone receptor (EcR) and ultraspiracle (USP), a retinoid X receptor (RXR) homolog (Kapitskaya et al., 1996; Hall and Thummel, 1998; Wang et al., 2000a, 2002). Upon binding 20E, the EcR/USP/20E receptor complex binds to ecdysone-response elements in the promoter region of target genes and activates transcription (Tran et al., 2001; Marquardt, 2005). Functional ecdysone-response elements are critical constituents of *Ae. aegypti YPP* gene promoters (Martin et al., 2001a). The ecdysone signal is subsequently amplified by a cascade of primary and secondary response genes that are activated by rising ecdysone levels (Spindler et al., 2009).

### How does 20E regulate YPP genes?—previtellogenic phase

During the previtellogenic state-of-arrest *YPP* gene expression is tightly repressed by multiple mechanisms, several of them affecting the reception of ecdysone signals. Both, EcR, and USP proteins are expressed during this stage in the *Ae. aegypti* female fat body (Yao et al., 1992; Wang et al., 2000b). However, these proteins do not form a heterodimer capable of binding to ecdysone response elements during this period. One key factor inhibiting the ecdysone hormonal cascade is the competitive binding of the orphan nuclear receptor AHR38 to USP during previtellogenesis (Zhu et al., 2000; Marquardt, 2005). After a blood meal and in the presence of 20E, EcR displaces AHR38 and forms a heterodimer with USP. Sevenup (Svp), a mosquito homolog of chicken ovalbumin upstream transcription factor, acts as a transcriptional repressor by binding directly to AGGTCA repeats within the ecdysone response elements of the vg-1 promoter, thereby inhibiting the ecdysone responsiveness of these promoters (Miura et al., 2002).

### How does 20E regulate YPP genes?—vitellogenesis

During the vitellogenic period, ecdysone is converted to 20E by hydroxylation within the cytoplasm of the fat body cells. It activates gene transcription within trophocytes directly by binding to the EcR/USP heterodimer. This complex then translocates into the nucleus and binds to ecdysone receptor response elements (Figure 2A) (Hall and Thummel, 1998; Wang et al., 1998). *Aedes aegypti* has two characterized EcR and USP isoforms (*Aa*EcRa and *Aa*EcRb AaUSPa and AaUSPb) (Kapitskaya et al., 1996; Wang et al., 2000a, 2002).

An orphan nuclear factor, β FTZ-F1, mediates fat body competence for 20E responsiveness. β FTZ-F1 is upregulated pre-and post-vitellogenesis and interacts with a p160/SRC type coactivator, FISC, to directly recruit EcR/USP/FISC to 20E promoters at the onset of the 20E signaling (Li et al., 2000; Raikhel et al., 2002; Zhu et al., 2006; Ou and King-Jones, 2013). Transcripts for another nuclear receptor, HR3, increase over the 24 h after a blood meal suggesting it also plays a role in 20E responsiveness (Kapitskaya et al., 2000). 20E activation of EcR/USP induces the early response genes, *E74*, *E75*, and *Broad*, which in turn, activate transcription of late target genes like *vg-1* to amplify the hormonal signal (Raikhel et al., 2002; Sun et al., 2004; Chen et al., 2005).

Interestingly, 20E is also found in the male accessory glands of *Anopheles gambiae* mosquitoes and large amounts are transferred to females during mating. This suggests that 20E plays a role in modulating post-mating effects in females. However, a role for male derived 20E in *YPP* gene regulation has not yet been demonstrated in this mosquito (Pondeville et al., 2008).

## Nutrient signaling

### What is the signal?

Mosquitoes ingest vertebrate blood which functions as a rich source of amino acids. Amino acids function as building blocks for yolk protein precursor synthesis and are also used for energy production (Marquardt, 2004). The bulk of the protein contained within vertebrate blood is hemoglobin which is digested into free amino acids. The amino acids are transported via the hemolymph to the tissues of the female mosquito. After a blood meal, free amino acid levels in the mosquito hemolymph rise sharply as hemoglobin digestion in the mosquito midgut proceeds (Marquardt, 2004).

### Mode-of-action

See Figure 2C The fat body monitors hemolymph amino acid levels via the target of rapamycin (TOR) signaling pathway which conducts the amino acid mediated signal to regulate *YPP* gene expression (Hansen et al., 2004). The key enzyme of this pathway, the TOR kinase, is a highly conserved protein that regulates protein translation in eukaryotic cells.

### How does the TOR signaling pathway regulate YPPs?

The exact mechanism eukaryotic cells utilize to sense hemolymph amino acid levels remains unknown. Amino acids are taken up by fat body trophocytes through a collection of specific amino acid transporter proteins that have different substrate specificities and may also work as receptors. The SLC7 family of cationic amino acid transporters play an important role in this process and are at the top of the nutrient signaling cascade in the fat body (Carpenter et al., 2012). RNAi-mediated gene knockdown of several of the SLC7-type transporters resulted in diminished TOR-signaling, *YPP* expression, and egg production (Attardo et al., 2006; Carpenter et al., 2012). The small GTPase Rheb functions upstream of TOR and is an indispensable part of this pathway in the fat body (Roy and Raikhel, 2011). Several downstream components of the TOR pathway are also associated with nutrient signaling in the fat body (Figure 2B). A well characterized downstream player in the TOR pathways is the S6 Kinase. S6 kinase is directly phosphorylated by TOR (Hansen et al., 2005) and in turn activates the translation of a GATA transcription factor in the fat body (AaGATAa). During the state-of-arrest, *YPP* gene transcription is repressed by another GATA transcription factor (*Aa*GATAr) that recognizes and binds to GATA-binding motifs upstream of the *YPP* gene region. Reception of a nutritional signal results in GATAr being displaced by GATAa upon the vg1 promoter. GATAa acts as a transcriptional activator which enhances *YPP* gene expression (Martin et al., 2001b; Attardo et al., 2003; Park et al., 2006). 4E-BP, a repressor of translation, is also a downstream target of TOR (Hay and Sonenberg, 2004). After a blood meal 4E-BP is hyper-phosphorylated in the mosquito fat body. However, treatment with the TOR inhibitor rapamycin does not block 4E-BP hyper-phosphorylation which indicates the involvement of other signaling pathways (Roy and Raikhel, 2012).

## Insulin-like peptide signaling

### What is it?

Insulin-like peptides (ILPs) are an evolutionary conserved group of peptide hormones which are characterized by a conserved disulfide bond structure (De Meyts, 2004). ILP signaling plays a crucial role in immunity, reproduction and longevity of mosquitoes (Luckhart and Riehle, 2007). There are eight ILPs and one insulin receptor described in *Ae. aegypti* (Graf et al., 1997; Riehle et al., 2006).

### Where are ILPs expressed?

ILPs and the insulin receptor are expressed in a variety of tissues in *Ae. aegypti* (Riehle and Brown, 2002; Riehle et al., 2006). Five ILPs are expressed in the brain and two are expressed in the head, thorax and abdomen of all life stages. These peptides assume a variety of possible roles including regulation of metabolic processes, cellular growth, lipid and glycogen processing, reproduction, and aging (Wu and Brown, 2006). Of the eight ILPs, ILP1, ILP3, and ILP 8 retain extremely conserved structures that are homologous to human insulin and relaxin 2 (Brown et al., 2008).

### ILPs receptor

All known insulin receptors have tyrosine kinase activity. The first arthropod insulin receptor was characterized in D. *melanogaster* (Wu and Brown, 2006). The one known mosquito ILP receptor bears high homology to vertebrate insulin receptors and is comprised of two subunits, alpha and beta (Graf et al., 1997; Riehle and Brown, 1999). The alpha subunit contains the insulin binding domain while the beta subunit contains a tyrosine kinase domain which is responsible for the phosphorylation of downstream targets. Administration of human and porcine insulin to mosquitoes is capable of activating ILP signaling via their endogenous insulin receptor (Roy et al., 2007; Pakpour et al., 2012).

### Mode of action

See Figure 2D Binding of the insulin receptor by ILPs activates a conserved signaling pathway as illustrated in Figure 2D (Wu and Brown, 2006). The phosphorylated insulin receptor substrate activates signaling via PI3K and other pathways. Downstream kinases phosphorylate effector proteins that regulate gene expression and other cellular processes.

### How does insulin regulate YPPs genes?

ILPs regulate *YPP* gene expression in the fat body both directly and indirectly. *In vitro* fat body culture experiments show that *YPP* expression is directly induced by the combination of the 20E and ILP signaling pathways acting together to activate gene expression. Roy et al., demonstrated the synergistic relationship of 20E and insulin by incubating isolated mosquito fat bodies for 3 h in different mixtures of porcine insulin and 20E (Roy et al., 2007). These experiments show that neither 20E nor insulin has an effect on expression individually; however a strong synergistic effect occurs when presented together.

ILPs also regulate *YPP* expression indirectly through the regulation of ecdysone synthesis. A blood meal stimulates the secretion of ILPs amongst other neuropeptides, namely OEH, that regulate ecdysteroid production (Riehle and Brown, 1999; Roy et al., 2007; Brown et al., 2008). In *Ae. aegypti*, ILP3 is characterized as the ILP responsible for initiating egg development (Brown et al., 2008). An experiment by Brown et al. demonstrates that ILP3, bovine, and porcine insulin stimulate the ovaries to produce ecdysteroids in decapitated females (Brown et al., 2008).

Manipulations of the ILP-signaling pathway in mosquitoes have resulted in profound changes in egg production. RNAi-mediated knockdown of the phosphatidylinositol-3,4,5-trisphosphate 3-phosphatase PTEN6, a suppressor of ILP signaling resulted in a significant increase in viable eggs produced by females (Arik et al., 2009).

One downstream effector gene of the ILP-signaling pathway has been characterized in mosquitoes. The forkhead-box transcription factor FOXO is a major target of ILP signaling in insects and vertebrates (Hwangbo et al., 2004). FOXO levels rise in the mosquito fat body after a blood meal and RNAi-mediated knockdown of this factor resulted in abolishment of *vg1* gene expression in isolated fat bodies that were stimulated with amino acids. Also, FOXO knockdown mosquitoes produced smaller egg numbers after a blood meal (Hansen et al., 2007). It is not known if this is due to direct or indirect effects.

## Crosstalk between pathways

The interplay between the different signaling pathways regulating YPP gene expression is complex and only partially understood. However, significant levels of crosstalk appear to occur between the aforementioned signaling pathways in the different organs and tissues of adult female mosquitos. Several studies address these complex interactions.

### JH/TOR

JH and nutrient signaling are connected in mosquitoes in two distinct ways. A recent study shows that JH biosynthesis is in part regulated by nutrient signaling via the TOR pathway (Perez-Hedo et al., 2013). Nutritional information impacts JH levels by regulating the expression of genes associated with the JH synthesis pathway in the corpora allata. A similar regulatory interplay between TOR signaling and JH expression was also found in the German cockroach, *Blatella germanica* (Maestro et al., 2009). JH in turn, regulates the expression of several genes coding for proteins associated with the TOR-signaling machinery in the mosquito fat body (Roy et al., 2007). Presence or absence of TOR pathway components profoundly influences the interpretation of nutritional signals by the fat body after a blood meal and decide if a blood meal results in immediate or delayed YPP synthesis.

### ILP3/TOR, ILP3/ecdysone, ILP/ecdysone/JH

ILP3 has emerged as a key regulator of vitellogenesis via multiple mechanisms. After a blood meal, ILP3 activates the expression of trypsin proteases in the midgut of mosquitoes thereby enhancing blood digestion and indirectly affecting TOR-mediated nutrient signaling (Gulia-Nuss et al., 2011). Three studies have demonstrated the effect of ILP signaling on ecdysone synthesis. The first study showed that unfed mosquitoes receiving ectopic treatment with bovine and porcine insulin results in activation of ecdysteroid secretion (Graf et al., 1997). The second study finds that ILP3 stimulates ecdysone synthesis and YPP uptake in female mosquitoes (Brown et al., 2008). The third study shows that insulin only stimulates ecdysteroid production when the mosquitoes are pre-treated with the JH analog methoprene (Borovsky et al., 1986).

A study in the red flour beetle, *Triboleum castaneum*, found that JH regulates *vg* gene expression through ILP signaling (Sheng et al., 2011). To our knowledge this has not been tested in mosquitoes, yet.

## Synopsis and outlook

Figure 2E shows a schematic of the organization of transcription factor binding sites in the *vg1* promoter of *Ae. aegypti*. Several signaling pathways converge in the regulation of these and other YPP genes. In recent years our understanding of the signaling pathways involved and the interplay between them has undergone rapid growth. We know that at least four distinct pathways are involved: juvenile hormone, ecdysone, insulin, and the TOR nutritional signaling pathway. The challenge for the future is to understand the crosstalk between these pathways in different organs and during different physiological stages in adult female mosquitoes. Based upon the current understanding of signaling in mosquito reproduction we feel that the ILPs may play a key regulatory role in the determination of autogenous vs. anautogenous life histories in mosquitoes.

In addition, while ecdysone- and juvenile hormone analogs are already used as insect growth regulators, a challenge for the future is to use the novel and detailed understanding of nutrient and insulin-signaling pathways to develop new strategies for mosquito control.

### Conflict of interest statement

The authors declare that the research was conducted in the absence of any commercial or financial relationships that could be construed as a potential conflict of interest.
